# A novel predicted model for hypertension based on a large cross-sectional study

**DOI:** 10.1038/s41598-020-64980-8

**Published:** 2020-06-30

**Authors:** Zhigang Ren, Benchen Rao, Siqi Xie, Ang Li, Lijun Wang, Guangying Cui, Tiantian Li, Hang Yan, Zujiang Yu, Suying Ding

**Affiliations:** 1 Department of Infectious Diseases, the First Affiliated Hospital of Zhengzhou University, Zhengzhou, 450052 China; 2 Health Management Center, the First Affiliated Hospital of Zhengzhou University, Zhengzhou, 450052 China; 3 Gene Hospital of Henan Province; Precision Medicine Center, the First Affiliated Hospital of Zhengzhou University, Zhengzhou, 450052 China

**Keywords:** Predictive markers, Risk factors

## Abstract

Hypertension is a global public health issue and leading risk for death and disability. It is urgent to search novel methods predicting hypertension. Herein, we chose 73158 samples of physical examiners in central China from June 2008 to June 2018. After strict exclusion processes, 33570 participants with hypertension and 35410 healthy controls were included. We randomly chose 70% samples as the train set and the remaining 30% as the test set. Clinical parameters including age, gender, height, weight, body mass index, triglyceride, total cholesterol, low-density lipoprotein, blood urea nitrogen, uric acid, and creatinine were significantly increased, while high-density lipoprotein was decreased in the hypertension group versus controls. Nine optimal markers were identified by a logistic regression model, and achieved AUC value of 76.52% in the train set and 75.81% in the test set for hypertension. In conclusions, this study is the first to establish predicted models for hypertension using the logistic regression model in Central China, which provide risk factors and novel prediction method to predict and prevent hypertension.

## Introduction

Hypertension which is a global public health issue is a leading risk for death and disability^1^. It exacerbates the burden of kidney failure, stroke, and heart disease, as well as premature mortality and disability. The number of people with hypertension worldwide increased by 400 million in 2008, and about 40% of adults over the age of 25 are diagnosed with hypertension. The risk of death associated with systolic blood pressure increases significantly with increasing systolic blood pressure^2^. Hypertension ranks first among all modifiable causes of cerebrovascular disease (CVD) and is the second preventable cause of death in the United States^3^. By 2030, the number of global CVD-related deaths will exceed 23.3 million^4^. It’s reported by the World Health Organization (WHO) that global CAD cause approximately 17 million deaths each year^5^, of which approximately 9.4 million die from complications of hypertension^6^. Hypertension accounts for at least 51% death of stroke and 45% death of heart diseases^5^. In 2010, hypertension has been the leading cause of disability-adjusted life years and death. Then, the disability-adjusted life year caused by CAD was 366 million in 2017. Among them, the disability-adjusted life year caused by hypertensive heart disease was 16.5 million^7^. The prevalence of hypertension in China was high (46.4%) in 2012–2015 according to the 2017 American College of Cardiology/American Heart Association guidelines^8^. However, the awareness, control and treatment of hypertension are unsatisfactory^8^. Thus, it is important and urgent to search a new method predicting hypertension.

The increased prevalence of hypertension is due to behavioral risk factors, aging and population growth, such as persistent stress, being overweight, lack of physical exercise, harmful consumption of alcohol and unhealthy diet^9^. The harmful effects of hypertension are complicated, including the increasing risk of kidney failure, stroke, and heart attack. In 1990, Pearson *et al*. firstly reported that they used cox proportional hazard regression to establish a predicted model for hypertension which was called the Johns Hopkins multiple risk equations^10^. Recent years, Chinese scholars conducted some prospective cohort studies and proposed that high uric acid level^11^, short sleep duration^12^ and high visceral adiposity index^13^ could enhance the incidence of hypertension. Then, some case-control studies indicated that certain genes^14,15^ and proteins^16^ were risk factors for hypertension. In this study, based on a large group of people, we analyzed the differences of clinical features between hypertension and non-hypertension patients, identified the risk factors of hypertension, and established a novel predicted model for hypertension. This study will provide risk factors and a novel prediction method to predict and prevent hypertension.

## Methods

### Participant information

This study was conducted in keeping with the Helsinki Declaration and Rules of Good Clinical Practice. The study was approved by the Institutional Review Board of the First Affiliated Hospital of Zhengzhou University (2017-XY-002). Written informed consents were signed by all participants at the time of registration. From June 2008 to June 2018, a total of 73158 participants from the Physical Examination Center, the First Affiliated Hospital of Zhengzhou University in Central China, were randomly enrolled. After removing 13 participants with outliers and 4165 participants with nulls values, the remaining 68980 participants were used for statistical analysis. Among them, 33570 participants with hypertension and 35410 participants without hypertension were included. Participants’ demographics and clinical information were collected from hospital electronic medical records (Supplementary Table 1).

### Definition of hypertension

According to the Guideline for the prevention, detection, evaluation, and management of high blood pressure in adults established by American Heart Association (AHA) and American College of Cardiology (ACC) On November 13, 2017, the range of normal BP was systolic blood pressure (SBP) < 120 mmHg and diastolic blood pressure (DBP) < 80 mmHg. The elevated BP was defined as SBP ranging 120~129 mmHg and DBP < 80 mmHg. Stage 1 hypertension was defined as SBP ranging 130~139 mmHg or DBP ranging 80~89 mmHg. Stage 2 hypertension was described as SBP ≥ 140 mmHg or DBP ≥ 90 mmHg^3^.

### Measurement method

The blood pressure of each participant was strictly measured twice at the house of the participants after a rest of 5 minutes by the trained interviewer who has learned standard method of AHA using standard mercury sphygmomanometer and appropriate-sized cuff according to a standard protocol to obtain an accurate value. The average of the two measurements was used for subsequent analysis.

### Biomarker identification and probability of disease (POD) construction

Firstly, we analyzed the differences of clinical features between the hypertension patients (case) and non-hypertension participants (control). Student T test was used for the significance test for all the phenotypes except for gender, and Chi-squared test was used for gender. A total of 12 phenotypes with significant difference (p value < 0.05) between case group and control group were selected for further analysis.

The POD index was defined as the ratio between the number of randomly generated decision trees that predicted sample as “hypertension” and that of the trees defining healthy controls. POD index of the train set was calculated using the optimal set of phenotypes and the receiver operating characteristic curve (ROC) was plotted (R 3.3.0, pROC package). The POD index of the test set, including 10596 healthy controls and 9980 hypertension patients, was calculated using the same optimal set of phenotypes.

Secondly, all data were randomly divided into the train set (70%) and the test set (30%) (Supplementary Table 2) by the “sample” function of R software. In the train set (n = 48404), logistic regression, using “glm” function R 3.4.1 software, was performed using the 12-phenotype value table of train set, including 24814 healthy controls and 23590 hypertension patients. Then Stepwise regression was used to obtain the optimal regression equation based on Akaike Information Criterion (AIC). 9-phenotype markers were chosen as the optimal set, if any one of them was dislodged, the AIC of the regression equation will be bigger. Probability of disease (POD) index of the train set was calculated using this set of phenotypes and a ROC was drawn (R 3.3.0, pROC package). The POD index of the other test set, including 10596 healthy controls and 9980 hypertension patients, was calculated using this set of phenotypes.Moreover, in the same train set, the Fivefold cross-validation was performed on a random forest model (R 3.4.1, randomForest 4.6–12 package) with default parameters except for ‘importance = TRUE’ using the 12 phenotypes in the train set, as previously described^17,18^. The cross-validation errors from five trials of the fivefold cross-validation were averaged. In addition, the cutoff was set by plus the minimum error in the averaged curve with the standard deviation at that point. All sets (≤7) of phenotype markers with an error less than the cutoff were listed, and the set with the smallest number of phenotypes was chosen as the optimal set.

### Statistical analysis

Statistical analyses were performed using R software. R software was used to constructed random forest model (R 3.4.1; random forest 4.6–12 package) and receiver operating characteristic (ROC) curve (R 3.4.1, pROC package). The student T test was used to evaluate the differences between the sets of continuous variables. The chi-square test was used to evaluate the differences between the sets of categorical variables.

## Results

**Figure 1:**
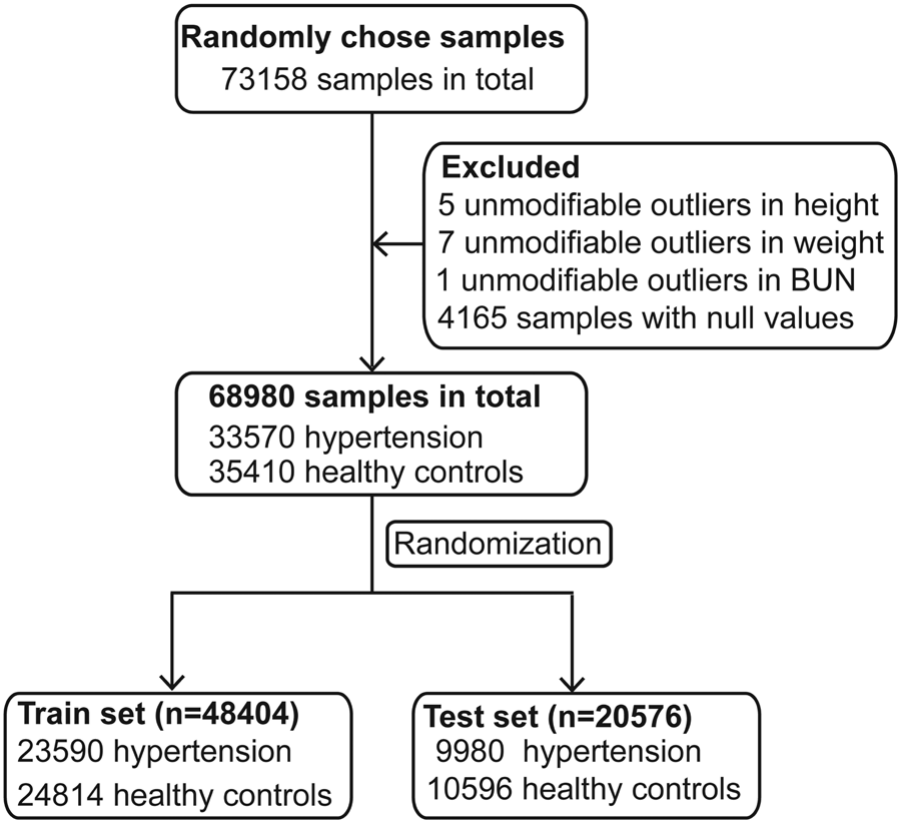
Study design and flow diagram. A total of 73158 participants were randomly enrolled. After removing 4165 participants with nulls values and 13 participants with outliers, the remaining 68980 participants, including 33570 participants and 35410 healthy controls, were used for further statistical analysis. We randomly chose 70% of the samples as the train set (n = 48404) and the remaining 30% as the test set (n = 20576) from both groups.

A total of 73158 participants were enrolled in this study. Then, 4165 participants with nulls values and 13 participants with outliers were excluded, and the remaining 68980 participants were divided into hypertension group (n = 33570) and healthy controls (n = 35410). We randomly chose 70% of the samples as the train set (n = 48404) and the remaining 30% of the samples as the test set (n = 20576) from both groups. The train set was used to constructed predicted model. Moreover, the predicted model was validated in the test set (Fig. 1).

### Clinical characteristics of participants

**Figure 2:**
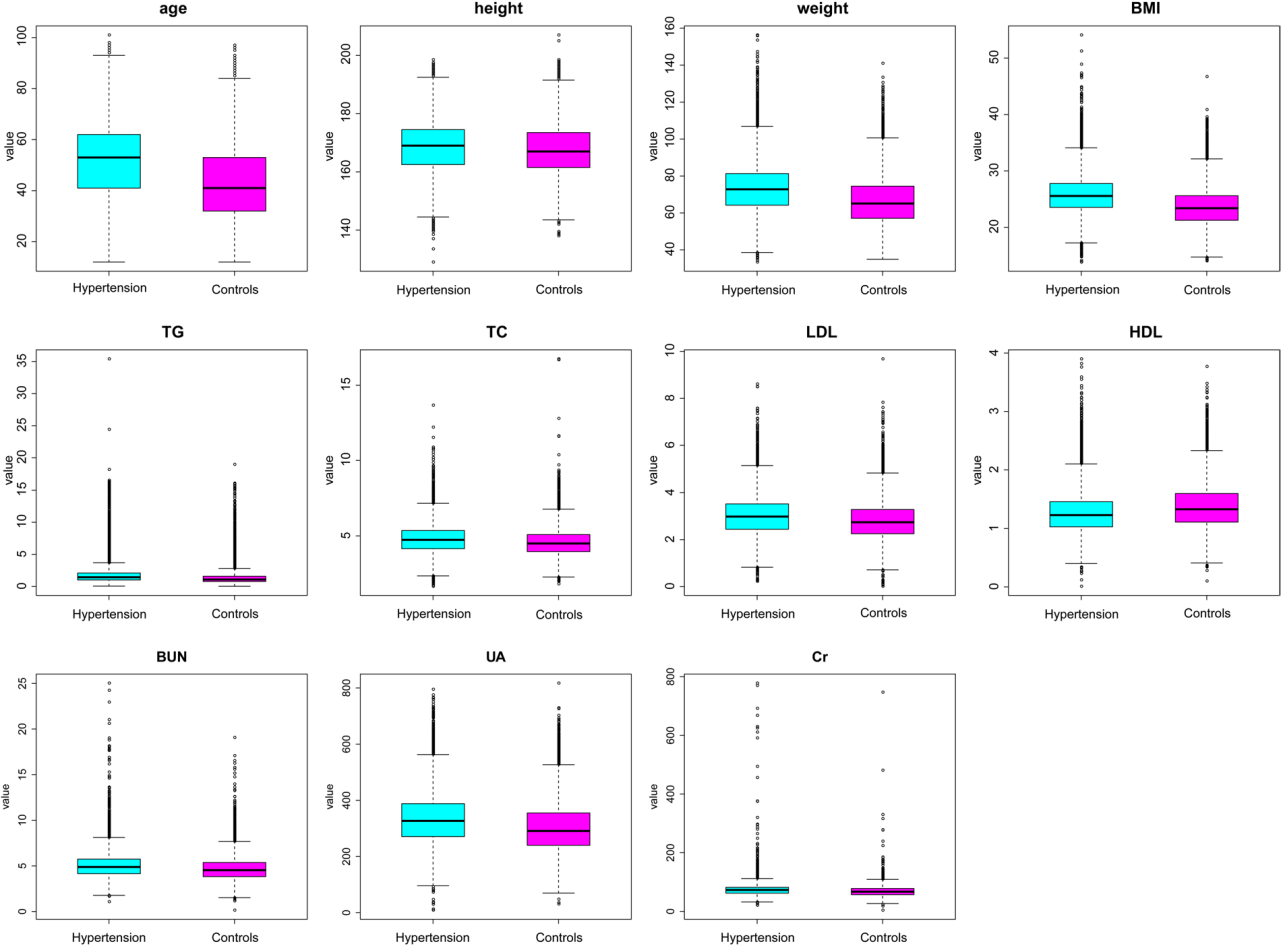
The difference and comparison of clinical parameters between the hypertension group (n = 33570) and healthy controls (n = 35410). The student T test was used to analyze the significant differences of clinical parameters between the hypertension group (case) and healthy controls (control). Clinical parameters included age, gender, height, weight, body mass index (BMI), triglyceride (TG), total cholesterol (TC), low-density lipoprotein (LDL), high-density lipoprotein (HDL), blood urea nitrogen (BUN), uric acid (UA), and creatinine (Cr).

Clinical characteristics of the participants, including age, gender, height, weight, BMI, triglyceride (TG), total cholesterol (TC), low-density lipoprotein (LDL), high-density lipoprotein (HDL), blood urea nitrogen (BUN), uric acid (UA), and creatinine (Cr), were analyzed between the hypertension group (n = 33570) and healthy controls (n = 35410) (Table 1, Fig. 2).

**Table 1:** Clinical characteristics of the enrolled participants.

Clinical indexes	Hypertension group (n = 33570)	Healthy controls (n = 35410)	P values
Age (year)	51.98 ± 14.77	42.72 ± 12.91	<2E-16
Gender			<2E-16
Female	9932 (35.71%)	17878 (64.29%)	
Male	23638 (57.42%)	17532 (42.58%)	
Height (cm)	168.35 ± 8.67	167.46 ± 8.13	4.02E-44
Weight (kg)	73.28 ± 12.90	66.36 ± 11.98	<2E-16
BMI	25.75 ± 3.39	23.56 ± 3.18	<2E-16
TG (mmol/L)	1.79 ± 1.43	1.35 ± 1.01	<2E-16
TC (mmol/L)	4.78 ± 0.95	4.57 ± 0.87	1.48E-208
LDL (mmol/L)	3.00 ± 0.83	2.80 ± 0.78	3.08E-231
HDL (mmol/L)	1.27 ± 0.35	1.38 ± 0.37	3.52E-302
BUN (mmol/L)	5.04 ± 1.32	4.67 ± 1.22	3.11E-312
UA (umol/L)	333.54 ± 87.69	301.77 ± 83.40	<2E-16
Cr (mmol/L)	72.92 ± 18.50	68.39 ± 15.08	1.84E-268

All data were presented as mean ± standard deviation (SD). The T test was used to evaluate the differences between the sets of continuous variables. The chi-square test was used to evaluate the differences between the sets of categorical variables. BMI, body mass index; TG, triglyceride; TC, serum total cholesterol; LDL, low-density lipoprotein; HDL, high-density lipoprotein; BUN, blood urea nitrogen; UA, uric acid; Cr, creatinine.

Compared to healthy controls, age level was significantly increased in patients with hypertension (p < 2E-16), suggesting that the older people are prone to suffer from hypertension. Meanwhile, male population presented an increased percentage of hypertension versus female population (p < 2.0 × 10^−16^). Also, BMI level was significantly elevated in the hypertension group versus healthy controls (p < 2.0 × 10^−16^), hinting that the fatty population are prone to present hypertension.

Moreover, serum levels of clinical parameters were measured. Compared to healthy controls, serum level of TG was markedly increased in the hypertension group (p < 2.0 × 10^−16^). Meanwhile, serum TC level was elevated in the hypertension group versus healthy controls (p = 1.48 × 10^−208^). Notably, serum LDL level was significantly increased (p = 3.08 × 10^−231^), but serum HDL was obviously decreased (p = 3.52 × 10^−302^), in the hypertension group versus healthy controls.

In addition, serum level of BUN was remarkably increased in the hypertension group versus healthy controls (p = 3.11 × 10^−312^). Compared with healthy controls, serum UA was significantly increased in the hypertension group (p < 2 × 10^−16^). Also, serum level of Cr was elevated in the hypertension group versus healthy controls (p = 1.84 × 10^−268^). Thus, these increased clinical parameters may become the independent risk factors for hypertension.

### Identification and validation of the predicted model for hypertension by a logistic regression model

**Figure 3:**
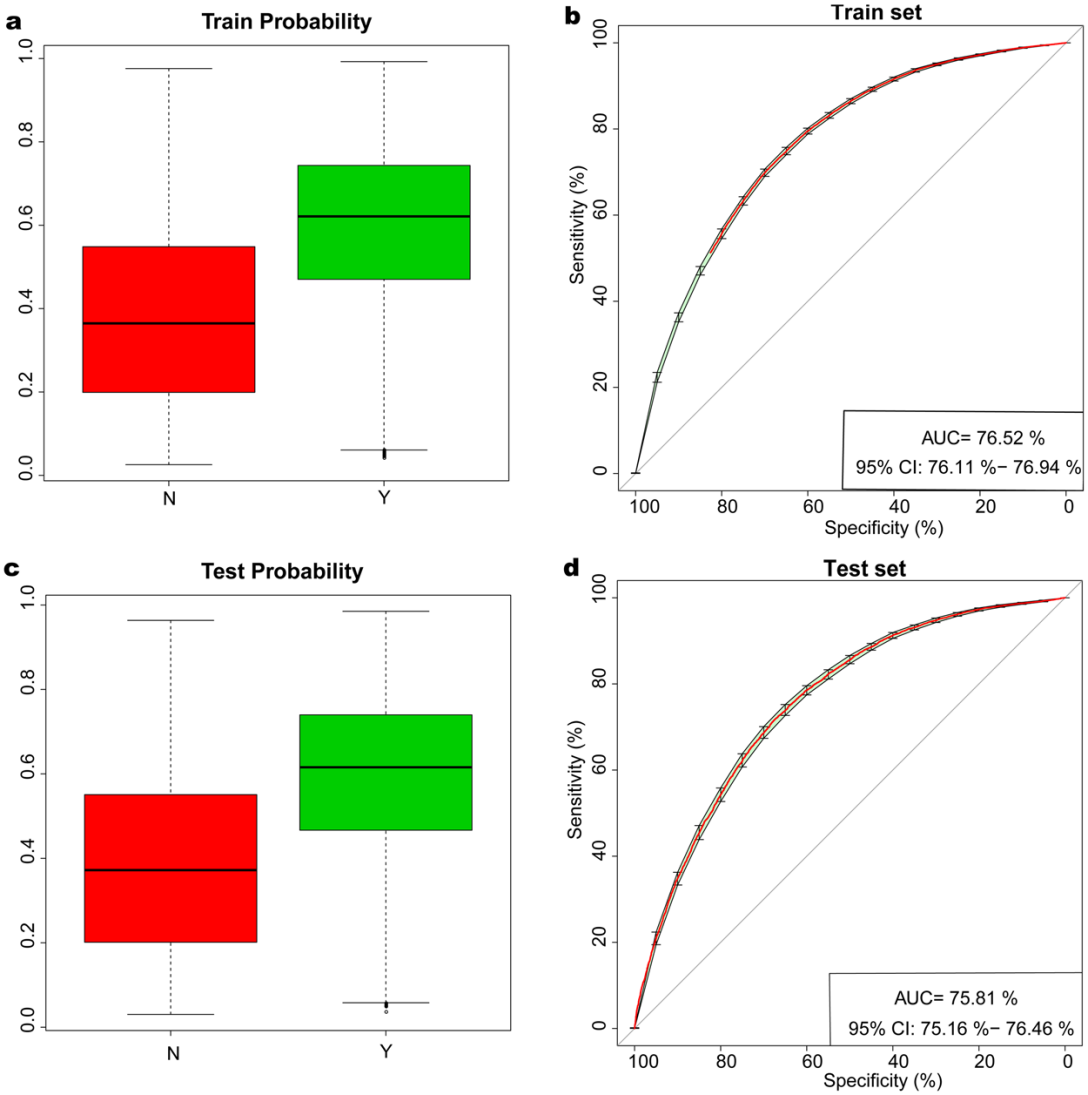
Identification and validation of the predicted model for hypertension by a logistic regression model. (**a**) The POD index was calculated based on the selected 9 optimal markers for each sample, and the mean POD value was significantly increased in the hypertension group versus healthy controls in the train set (p < 0.001). (**b**) The AUC value of the POD index achieved 76.52% (95% CI: 76.11–76.94%) between the hypertension group and healthy controls in the train set. (**c**) The mean POD value was significantly increased in the hypertension group versus healthy controls in the test set (p < 0.001). (**d**) The AUC value of the POD index achieved 75.81% (95% CI: 75.16–76.46%) between the hypertension group and healthy controls in the test set.

To establish the predicted model for hypertension, we randomly chose 70% of the samples as the train set (n = 48404) and the remaining 30% of the samples as the test set (n = 20576) from both groups. In the train set, we identified the 9 optimal distinguishing markers, including gender, age, height, weight, TG, LDL, HDL, UA and Cr, for hypertension through a logistic regression model (Table 2). The POD index was calculated based on the selected 9 optimal markers for each sample, and the mean POD value was significantly increased in the hypertension group versus healthy controls in the train set (p < 0.001, Fig. 3a). The AUC value of the POD index achieved 76.52% (95% CI: 76.11–76.94%) between the hypertension group and healthy controls in the train set (Fig. 3b). Furthermore, in the test set, the mean POD value was significantly increased in the hypertension group versus healthy controls (p < 0.001, Fig. 3c). Correspondingly, the AUC value of the POD index achieved 75.81% (95% CI: 75.16–76.46%) between the hypertension group and healthy controls (Fig. 3d). These results demonstrated that the predicted model for hypertension could distinguish the hypertension from healthy controls based the logistic regression model.

**Table 2:** Logistic regression model in the train set.

	Estimate	Std. Error	Z value	P value
(Intercept)	−0.30624	0.325476	−0.941	0.347
Gender	0.681627	0.034855	19.556	<2E-16
Age	0.04933	0.00083	59.463	<2E-16
Height	−0.04423	0.002062	−21.445	<2E-16
Weight	0.054823	0.001293	42.4	<2E-16
TG	0.15022	0.010256	14.648	<2E-16
LDL	0.106544	0.013039	8.171	3.05E-16
HDL	0.311486	0.034023	9.155	<2E-16
UA	0.001137	0.000157	7.225	5.00E-13
Cr	−0.00323	0.000793	−4.077	4.55E-05

The 9 optimal distinguishing markers, including gender, age, height, weight, TG, LDL, HDL, UA and Cr for hypertension were identified through a logistic regression model in the train set. TG, triglyceride; LDL, low-density lipoprotein; HDL, high-density lipoprotein; UA, uric acid; Cr, creatinine.

### Identification and validation of phenotype markers for hypertension by random forest model

To establish a more effective predicted model for hypertension, in addition to the logistic regression model, we also chose a random forest model with a fivefold cross-validation (Supplementary Figure 1). All analyses were based on the same train set and test set. The mean POD index was significantly increased in the hypertension group versus healthy controls. The AUC value of the POD index achieved 75.33% (95% CI: 74.9–75.76%) in the train and 74.65% (95% CI: 73.99–75.32%) in the test set.

To sum up, these results demonstrated that the logistic regression model achieved a better efficacy than the random forest model because of the higher AUC value.

## Discussion

According to a recent global overview of hypertension published by the WHO, hypertension has become a global health burden and a global public health crisis^19^. The burden cardiovascular disease including hypertension in China is increasing along with urbanization, rising incomes, and ageing of the population^20,21^. Hypertension is the leading modifiable risk factor for cardiovascular disease, which represents the top cause of death in China^21,22^. The prevalence of hypertension has been on the rise over the years^3,8,23^. Moreover, some studies have shown that the rates of undiagnosed hypertension were also high in many countries^24^. The establishment of the predicted model for hypertension helps prevent and improve high blood pressure. Therefore, this study conducted a random survey and analysis of 73158 participants in the Central China. We firstly analyzed the differences of clinical characteristics between the hypertension group (n = 33570) and healthy controls (n = 35410), and found that clinical parameters including age, height, weight, BMI, TG, TC, LDL, BUN, UA and Cr, were significantly increased, while HDL was decreased in the hypertension group versus healthy controls. Furthermore, to establish an effective predicted model for hypertension, we randomly chose 70% of the samples as the train set and the remaining 30% of the samples as the test set. In the train set, we identified the optimal distinguishing markers and established the POD index for hypertension. By random forest model, the POD index achieved 75.33% of AUC value (95% CI: 74.9–75.76%) in the train set, and achieved 74.65% of AUC value (95% CI: 73.99–75.32%) in the test set. Moreover, by a logistic regression model, the AUC value of the POD index achieved 76.52% (95% CI: 76.11–76.94%) in the train set, and achieved 75.81% (95% CI: 75.16–76.46%) in the test set. Thus, the predicted model for hypertension achieved a better efficacy by a logistic regression model. These findings provide a novel predicted method for hypertension, and benefit for the prevention of hypertension.

More and more clinical studies established predicted models based on big data in recent years^17,25^. In China, Zhang *et al*.^26^ analyzed data from 26,496 hypertensive patients and they set up a hypertension synthetic predictor by weighing the risk of hypertension for each factor to build a risk assessment matrix with an AUC of 0.755 for men and 0.800 for women. Both Chen *et al*.^27^ and Qi *et al*.^28^ analyzed the state of hypertension in northern China and established predicted models from different perspectives. Chen *et al*. established models using traditional risk factors, including BMI, age, SBP, DBP, serum glucose level and alcohol consumption for both women and men, while current smoking, neutrophil granulocyte, and total cholesterol only for women (AUC = 0.753), gamma-glutamyltransferaseonly for men (AUC = 0.761). Whereas Qi *et al*. conducted a large blood pressure genome-wide association study and used genetic risk score as the prediction factors. They evaluated the differences of genotype between the patients and healthy controls, and they found that differences were significant for rs17249754, rs11191548 and rs35444. The further application indicated that the chance of developing hypertension increased as the risk score increased by the genetic risk scoring method. There are also many similar studies in the US^10,29–32^ and Europe^33–37^. Different predicted models are available for different regions and their prediction power needs to be validated in large cohorts. Thus, we conducted a study on a large group of 73,158 participants that included 33750 patients with hypertension and 35410 healthy controls. We identified the optimal distinguishing markers and established the POD index for the predicted model for hypertension by random forest model and logistic regression model between the hypertension group and healthy controls. Moreover, we got high AUC values of 75.33% in the train set and 74.65% in the test set which indicated that the predicted model for hypertension could efficiently distinguish the hypertension from healthy controls based on the random forest model. Meanwhile, to establish a more effective predicted model for hypertension, we further analyze clinical parameters by a logistic regression model based on the same train set and test set which got higher AUC values (76.52% for the train set and 75.81% for the test set). A higher AUC value meant that the predicted model for hypertension established by the logistic regression model was more effective.

We constructed predicted models by establishing random forest model and logistic regression model, in which we identified independent risk factors for hypertension.There are significant differences in the risk factors, prevalence and distribution of hypertension in different regions. The risk factors we identified from random forest model were gender, age, height, weight, BMI, TG, and TC. In addition, we conducted a logistic regression model to get nine independent risk factors, including gender, age, height, weight, TG, LDL, HDL, UA, and Cr. The WHO pointed out that the global increase in the prevalence of hypertension was attributed to exposure to persistent stress, excess weight, lack of physical activity, harmful use of alcohol and unhealthy diet^19^. Clinical studies showed a significant relationship between ageing and increased blood pressure, with advancing age being a major immutable risk factor in the progreess of hypertension^38–41^. The Nurses’ Health Study II, which organized by Forman JP *et al*. was a prospective cohort study of 116671 female nurses since 1991. In this large-scale prospective study of women, they identified six low-risk lifestyle factors such as engaging in vigorous physical exercise, avoiding nonnarcotic analgesics, taking supplemental folic acid and drinking a little alcohol and so on. These factors were significantly associated with the decline in the prevalence of hypertension^42^. In 2015, Ying *et al*. published an article about the relationship between BMI and hypertension in the Lancet magazine. The analyses of the study based on data from 135715 individuals. Findings showed lower BMI reduced the risk of developing hypertension^43^. In recent years, a number of studies have shown that TG, HDL, LDL and TG can be used as predictors of essential hypertension events by constructing different models^44–49^. Fottrell *et al*. surveyed a random sample of 12,280 adults >30 years old in 96 villages in rural Bangladesh and found that obesity, smoking, passive smoking and increased salt consumption were risk factors for hypertension^50^. Kidney diseases can lead to elevated levels of urinary albumin, while micro albuminuria is considered as a risk factor for hypertension^51,52^. In recent years, there have been more and more studies on the ratio of urinary albumin-creatinine. On the one hand, the higher normal urinary albumin-creatinine ratio is significantly associated with an increased risk of hypertension^53,54^. On the other hand, a urinary albumin/creatinine ratio of less than 30 mg/g is a predictor of hypertension and cardiovascular mortality^55^. However, studies on creatinine as an independent risk factor for hypertension are rare. In 2015, Wu *et al*. from Jilin University studied the hypertension in Northeast China. He found that the risk factors for hypertension in Northeast China were age, gender, BMI, smoking, and drinking^56^. However, we analyzed data from 73158 participants in the Central China, and found that the independent risk factors for hypertension were gender, age, height, weight, TG, LDL, HDL, UA, and Cr. The result confirmed that there were significant differences in the risk factors of hypertension in different regions.

In the experiment, we used train set and test set to ensure the authenticity and effectiveness of the result. After excluding some outliers and null values, we randomly chose 70% of each of the two groups as the train group (23590 from hypertension group and 24814 from non-hypertension group). We used the remaining 30% of the samples as the test group (9980 from hypertension group and 10596 from non-hypertension group). Nine optimal markers were identified by a logistic regression model, and achieved AUC value of 76.52% in the train set and 75.81% in the test set for hypertension.

This study was the first to describe the characteristics of hypertension in the Central China. We also found independent risk factors for hypertension while constructing a predicted model of hypertension. This study provided data on risk factors affecting prevalence of hypertension in the Central China. It may guide the development of practical and effective strategies for managing and preventing hypertension. Meanwhile, it also provides reference for the management and prevention of hypertension in the world.

## Supplementary information

Supplementary Figure 1
