# Supplementary material for: A novel predicted model for hypertension based on a large cross-sectional study
Source: Sci Rep. 2020 Jun 30;10:10615. doi: 10.1038/s41598-020-64980-8 (PMC7327010; doi:10.1038/s41598-020-64980-8)
Supplement: Supplementary file 1 — Supplementary Figure 1 [file 41598_2020_64980_MOESM1_ESM.docx]

**Table S. The distribution information of variables in the 1199 samples.**

| **variable** | **mean(std)** | **median (IQR)** | **min** | **max** | **missing number** | **missing rate** | **tag** |
| --- | --- | --- | --- | --- | --- | --- | --- |
| age | 35.2 (16.4) | 34.0 (24.0-47.0) | 1.0 | 90.0 | 13 | 1.1 | impute |
| LOS | 7.3 (6.9) | 5.0 (2.0-11.0) | 1.0 | 42.0 | 38 | 3.1 | impute |
| ingestion_volume | 60.6 (69.8) | 30.0 (15.0-80.0) | 0.5 | 500.0 | 105 | 8.6 | impute |
| N1 | 83.8 (13.6) | 89.1 (79.4-92.5) | 5.1 | 98.3 | 75 | 6.1 | impute |
| WBC1 | 14.0 (8.1) | 11.8 (8.5-17.0) | 2.8 | 93.0 | 77 | 6.3 | impute |
| AST1 | 52.5 (141.3) | 23.0 (17.0-34.0) | 1.0 | 2025.0 | 84 | 6.9 | impute |
| ALT1 | 42.8 (117.0) | 17.0 (12.0-29.0) | 2.0 | 2336.0 | 90 | 7.4 | impute |
| GGT1 | 47.0 (100.7) | 18.5 (13.3-33.2) | 2.0 | 1274.0 | 146 | 11.9 | impute |
| CK_MB1 | 27.6 (39.7) | 17.8 (12.3-30.0) | 0.6 | 617.6 | 162 | 13.3 | impute |
| BUN1 | 6.8 (7.7) | 5.0 (3.9-7.0) | 1.4 | 178.0 | 95 | 7.8 | impute |
| CR_1 | 106.1(128.5) | 63.6 (50.0-102.0) | 17.0 | 1633.0 | 94 | 7.7 | impute |
| MPV | 9.2 (3.7) | 8.9 (8.0-9.9) | 0.8 | 90.8 | 77 | 6.3 | impute |
| PLT | 179.3 (92.0) | 171.0 (110.0-240.0) | 0.1 | 641.0 | 77 | 6.3 | impute |
| PCT | 0.2 (0.1) | 0.2 (0.1-0.2) | 0.0 | 0.6 | 83 | 6.8 | impute |
| LDH1 | 273.6(194.1) | 217.0 (177.0-294.0) | 61.0 | 2224.0 | 148 | 12.9 | impute |
| DBiL1 | 9.4 (22.8) | 4.9 (3.3-6.7) | 0.4 | 288.4 | 98 | 8.0 | impute |
| CK1 | 281.1(1287.2) | 111.0 (72.0-182.5) | 0.0 | 32034.0 | 214 | 17.5 | delete |
| PaCO2_1 | 31.6 (8.4) | 31.9 (26.3-36.7) | 7.4 | 77.0 | 668 | 54.7 | delete |
| Amylase_1 | 228.6 (425.9) | 93.0 (55.0-198.0) | 4.0 | 3557.0 | 425 | 34.8 | delete |
| Lipase_1 | 139.0 (441.8) | 24.3 (17.2-44.0) | 1.6 | 6222.0 | 459 | 37.6 | delete |
| PaCO2 | 31.5 (8.4) | 31.9 (26.2-36.6) | 7.4 | 77.0 | 656 | 53.7 | delete |
| PQ_concentration | 151.4 (877.7) | 3.3 (0.2-46.6) | 0.0 | 24864.0 | 240 | 19.6 | delete |
| CD3 | 651.8 (664.3) | 449.5 (301.0-785.0) | 63.0 | 4944.0 | 1074 | 87.9 | delete |
| CD4 | 331.8 (348.1) | 224.0 (144.0-370.0) | 32.0 | 2544.0 | 994 | 87.0 | delete |
| CD8 | 282.3 (307.0) | 196.0 (128.0-331.0) | 24.0 | 2304.0 | 994 | 87.0 | delete |
| IgM1 | 1.2 (0.6) | 1.0 (0.8-1.2) | 0.5 | 3.1 | 1112 | 97.3 | delete |
| IgA1 | 1.1 (0.6) | 0.9 (0.7-1.5) | 0.4 | 2.5 | 1112 | 97.3 | delete |
| IgG1 | 8.3 (2.4) | 8.6 (6.5-9.4) | 4.8 | 17.4 | 1112 | 97.3 | delete |
| C3_1 | 1.0 (0.2) | 1.0 (0.9-1.1) | 0.5 | 1.7 | 1110 | 97.1 | delete |
| C4_1 | 0.2 (0.1) | 0.2 (0.2-0.3) | 0.1 | 0.8 | 1110 | 97.1 | delete |
| TBiL1 | 17.1 (20.0) | 12.3 (8.4-17.9) | 0.6 | 304.9 | 288 | 23.6 | delete |
| cTnI1 | 0.0 (0.2) | 0.0 (0.0-0.0) | 0.0 | 1.8 | 951 | 77.8 | delete |
| cystain_C | 7.3 (3.7) | 8.4 (6.6-9.6) | 0.3 | 15.6 | 525 | 43.0 | delete |
| PH_1 | 7.4 (0.2) | 7.4 (7.3-7.4) | 6.8 | 9.6 | 681 | 55.7 | delete |
| Pao2_1 | 83.6 (27.3) | 84.0 (65.3-101.0) | 11.0 | 188.0 | 682 | 55.8 | delete |
| LAC1 | 4.1 (4.0) | 2.6 (1.5-4.9) | 0.3 | 26.0 | 700 | 57.3 | delete |
| HCO3_1 | 18.7 (6.2) | 19.7 (14.2-23.1) | 2.1 | 41.8 | 700 | 57.3 | delete |

**Figure S.** **The relationship of in-hospital death and variables**

**
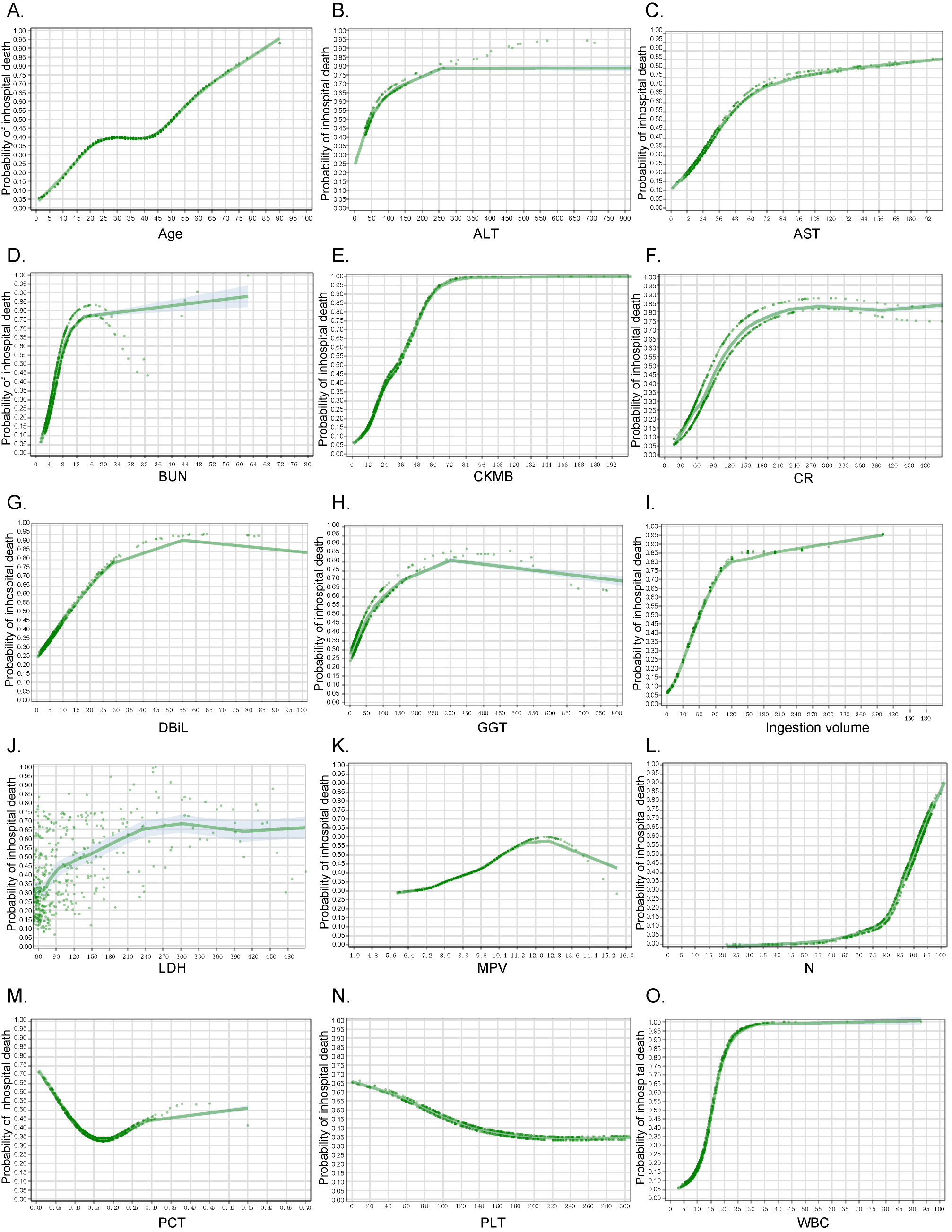
**
